# The Lived Experiences of Racial and Ethnic Minority Nurses Exposed to Racial Microaggressions in the Hospital Setting: Qualitative Study

**DOI:** 10.2196/67029

**Published:** 2025-06-18

**Authors:** Da S Kim, Ha Do Byon

**Affiliations:** 1 School of Nursing, The University of Virginia, 202 Jeanette Lancaster Way, PO Box 800782, Charlottesville, VA, 22903, United States, 1 434-243-3973; 2 MedStar Georgetown University Hospital, Washington, DC, United States

**Keywords:** nurse, ethnic, minority, marginalized, violence, workplace, microaggression, racism, discrimination, diversity, inclusion, belonging, nurse-patient relationship, Asian, Latinx, African American, Middle Eastern, qualitative, perspective, attitude, lived experience, interview, COVID-19, SARS-CoV-2, coronavirus, respiratory, infectious, pulmonary, pandemic

## Abstract

**Background:**

Type II (client-on-worker) workplace violence (WPV) between patients and nurses is an ongoing safety and health challenge in health care. However, little is known about the experiences of racial and ethnic minority nurses specifically in a profession in which most individuals identify as White. During and after the COVID-19 pandemic, type II WPV against certain minority groups increased, which suggests that underrepresented racial and ethnic minority nurses may have unique experiences with type II WPV inflicted by patients, their family members, or visitors.

**Objective:**

The aim of this study was to (1) explore the lived experiences of racial and ethnic minority nurses who have faced type II WPV from patients in the hospital setting, and (2) assess the emotional and physical effects of type II violence among racial and ethnic minority nurses.

**Methods:**

Semistructured individual interviews were conducted with racial and ethnic minority nurses. The research team recruited participants through snowball sampling. Nurses were eligible to participate if they (1) were ages 18 years and older, (2) were currently working as a registered nurse in a hospital in the United States or had previous experience in this role, with the experience dating no earlier than March 2020, when the COVID-19 pandemic began, (3) had experienced WPV from patients, their family members, or visitors at some point during their career, and (4) identified as a racial and ethnic minority. Interviews were conducted between February 2023 and March 2023. A qualitative descriptive approach was used to analyze the findings.

**Results:**

A total of 10 nurses from racial and ethnic minority groups were interviewed: 5 Asian, 2 Latina, 2 African American, and 1 Middle Eastern nurse. Violence experienced by the nurses fell under 2 categories: macroaggressions and microaggressions. Macroaggressions included physical violence, verbal abuse, and sexual violence. Microaggressions were subtle and often unconscious and unintentional comments, interactions, or behaviors relating to the participants’ race. All nurses (10/10) reported experiencing racial microaggressions and considered them very harmful. Microaggressions left a negative impact on these nurses in terms of their self-esteem, the nurse-patient relationship, and their job performance. However, many participants did not speak up about microaggressions to either the perpetrator or management because they feared that their experiences would be dismissed. Minimization and normalization of microaggressions were common themes among participants. In total, 90% of participants (9/10) expressed that they do not feel supported in the hospital as nurses of underrepresented minority groups.

**Conclusions:**

Microaggressions are a form of WPV. “Micro” implies small, but the consequences of microaggressions are additive and detrimental. Racial microaggressions negatively impact nurses in terms of their personal well-being, job performance, and ability to deliver quality patient care. Given this, more policies, procedures, and resources must be in place to support racial and ethnic minority nurses in the hospital setting.

## Introduction

### Background

According to the University of Iowa Injury Prevention Research Center, workplace violence (WPV) is divided into 4 main categories: Type I, Type II, Type III, and Type IV [1]. Type II (client-on-worker) WPV is the most common in health care settings [2], involving violence committed by patients, their family members, or visitors.

Compared to other health care professionals, nurses in particular are at a higher risk of being abused in the workplace since they spend more time with patients [3]. Thus, type II WPV is a prevalent issue between nurses and patients in hospitals. In fact, according to a survey conducted in 2024 by National Nurses United, 8 in 10 nurses (81.6%) have experienced at least one type of WPV in the past year [4]. WPV has been found to result in psychological consequences among nurses, including increased levels of anxiety, powerlessness, and helplessness, as well as manifestations of burnout such as emotional exhaustion, diminished job satisfaction, and decreased patient safety [3]. Research has been conducted to describe the overall prevalence and negative effects of WPV among nurses, but unfortunately, little is known about the experiences of nurses from racial and ethnic minority groups specifically in what is a profession in which most individuals identify as White. In 2020, about 81% of nurses were White; in 2022, 80% of nurses were White [5].

The COVID-19 pandemic has shed more light on the experiences of WPV among racial and ethnic minority nurses. Between March 2020—the beginning of the COVID-19 pandemic—and April 2022, there was a 119% increase in WPV among hospital nurses as a whole across all 50 states in the United States, with most assaulters being patients [6]. But Asian American workers specifically were left in a painful position during the pandemic, as there was an increase in anti-Asian racism, violence, and xenophobia [7]. Asian nurses in Canada and the United States actually admitted to experiencing more racism from colleagues, patients, and patients’ family members during the pandemic compared to pre-pandemic [8]. They experienced microaggressions such as questions about their ethnic origin and health status with COVID-19, derogatory comments on Asian stereotypes, and blatant discrimination (ie, rejection of care and racial profanities) [8]. A scoping review further confirmed that the COVID-19 pandemic exacerbated racial discrimination against nurses from racial and ethnic minority groups [9].

These findings suggest that nurses from racial and ethnic minority groups may have unique experiences with WPV. We aimed to further explore their experiences.

### Literature Review

Our literature review revealed a lack of studies understanding WPV among nurses from racial and ethnic minority groups. We performed an extensive search on 3 databases focused on health care: CINAHL, PubMed, and Web of Science. We were strictly interested in qualitative studies that explored experiences with WPV among bedside registered nurses from racial and ethnic minority groups. The initial search included the terms “nurses,” “violence,” “harassment,” “hospitals,” and “qualitative.” From here, we identified potentially relevant articles based on title and abstract screening and eliminated articles that were duplicates or not relevant based on full-text review. Articles that were excluded were: published before the year 2020 (the start of the COVID-19 pandemic), conducted outside of North America, systemic reviews, and focused on the experiences of non-bedside registered nurses, such as nursing students, physicians, advanced practice nurses (ie, nurse practitioners), physician assistants, and nurse managers. Our literature review resulted in 1 relevant qualitative study conducted in Canada.

The study found that it was uncommon for White nurses to be discriminated against by patients, and discrimination was mostly reported by nurses from racial and ethnic minority groups [10]. In addition, compared to their White counterparts, racial and ethnic minority nurses experienced sexual harassment, racial slurs, disrespect, rejection or questioning of care by patients for perceived incompetence, degradation, and abuse because of skin color, language barriers, or accents [10]. Nurses from racial and ethnic minority groups reported feeling stereotyped because they were seen to be of a lower class [10].

There have been high rates of violence and aggression experienced by racial and ethnic minority nurses. However, few studies have been conducted to understand the thoughts, feelings, and perceptions of racial and ethnic minority nurses in relation to these experiences. Such studies are needed because there may be unique emotional and physical imprints that WPV and aggression leave on nurses of underrepresented backgrounds.

### Goal of This Study

By gaining a better understanding of the experiences of nurses from ethnic and minority groups, hospitals can tailor resources to best meet their unique needs and cultivate a safer environment where they feel seen, heard, and understood. Given this, the purposes of this study were to (1) explore the lived experiences of racial and ethnic minority nurses who have faced type II WPV in the hospital setting and (2) assess the emotional and physical effects of type II violence among nurses from racial and ethnic minority groups.

## Methods

### Study Design and Recruitment

To explore the experiences of nurses from racial and ethnic minority groups with type II WPV in the hospital setting, we conducted a qualitative descriptive study, following the methodology outlined by Colorafi and Evans [11]. Qualitative description is grounded in the principles of naturalistic inquiry and the concept of truth. It does not require deep theoretical inference or interpretation. Rather, it focuses on studying the topic in a way that is straightforward and avoids deep theoretical abstraction. Data are described and organized in their natural state, through lower levels of inference and manipulation. This approach leads to “true understanding” of the material [11]. This study used the qualitative descriptive approach to ensure that participants’ experiences could be studied and described as closely as possible to how they were shared. We aimed to honor the authenticity of the participants’ voices and ultimately provide a straightforward and comprehensive summary of findings.

Nurses were eligible to participate in this study if they (1) were ages 18 years and older, (2) were currently working as a registered nurse in a hospital in the United States or had previous experience in this role, with the experience dating no earlier than March 2020, when the COVID-19 pandemic began, (3) had experienced type II WPV from patients, their family members, or visitors at some point during their career, and (4) identified as a racial and ethnic minority. WPV was defined broadly, including, but not limited to: verbal abuse, physical assaults, sexual assaults, threats of harm, harassment, microaggressions, etc.

### Ethical Considerations

The study was approved by the Institutional Review Board of the University of Virginia (#5532). Prior to initiating the interviews, all participants were informed about the study’s objectives, methods, potential risks, benefits, and data protection measures. Consent was obtained through Qualtrics, a secure online survey platform. No direct identifiers were collected, and all data were collected anonymously. The only information collected from participants were race and ethnicity. No additional data (eg, name, gender, age) were collected to protect participant confidentiality. Participants did not receive compensation for this study.

### Data Collection

Semistructured individual interviews were conducted with registered racial and ethnic minority nurses using snowball sampling (N=10) between February 2023 and March of 2023. The sample size was determined by data saturation [12]. After the tenth interview, the ethnic and racial breakdown of participants was diverse, and the first and second author determined that additional data collection would no longer contribute to new insights, patterns, or themes. Because the participants in this study were interviewed based on specific eligibility requirements, their perspectives were rich enough to reach saturation after 10 interviews. Semistructured individual interviews were the preferred method of data collection to gather more open-ended data and delve deeper into experiences that may have been sensitive for the participants. Interviews were conducted by the first author (DSK) through one-on-one Zoom video sessions. Multimedia Appendix 1 shows the semistructured interview guide that was used. Opener questions were posed to participants at the beginning of the interview to create a more comfortable environment, such as: “What race/ethnicity best describes you?” “Tell me about your job;” “Tell me what your typical work day looks like;” “Tell me about your interactions with your patients, their family members, or visitors.” Subsequent questions were aimed at exploring the participant’s experiences with type II WPV. These questions included: “Tell me about any situation where you experienced workplace violence from patients, their family members, or visitors during your time as a nurse;” “How did you feel when you experienced this workplace violence?” “What thoughts occur to you when you experience violence?” “In what ways, if any, does workplace violence affect your job performance?” Interviews typically lasted between 60 and 90 minutes. At the end of the interview, the participant was provided with a list of resources relating to mental health and WPV due to the vulnerable and sensitive nature of the topic.

### Data Analysis

Data analysis began after all interviews were completed. The data was organized following Colorafi and Evan’s [11] descriptive qualitative framework and conventional content analysis, a type of analysis that describes a phenomenon where existing research and theory are limited. Recorded interviews were transcribed verbatim by the first author (DSK). The transcripts were read closely multiple times to develop a deep understanding of the data. Sentences or paragraphs from the transcripts were divided into meaning units: segments of text that contain a single idea [11]. Descriptive codes were applied to each meaning unit, and these codes were then grouped into broader categories or themes based on conceptual similarities. The analysis was an interactive process, where codes and themes were continuously reviewed, refined, and revisited as analysis progressed. Codes and themes were developed by the first author (DSK) and finalized with the second author (HDB). There were no concerns or disagreements regarding the codes and themes.

Throughout the entire process of data analysis, we used Colorafi and Evan’s [11] 5 standards to ensure trustworthiness of the study: objectivity, dependability, credibility, transferability, and application.

To ensure objectivity, we avoided inflicting personal assumptions and bias and described the study’s methods and procedures in detail. To ensure dependability, we maintained consistency in procedures by adhering to Colorafi and Evan’s [11] qualitative descriptive analysis method and referencing an interview guide across all participants during interviews. To ensure credibility, the data analysis was reviewed by the second author to confirm that the first author’s discovered themes accurately aligned with participants’ experiences. To ensure transferability, we provided detailed characteristics of participants (ie, race and ethnicity, role as hospital nurses). We also provided thick descriptions through direct quotes from participants’ interviews and rich details of their experiences. Finally, to ensure application, we have suggested ways to stimulate further research under the “Future Research” section of this paper.

## Results

### Interviewee Characteristics and Their Experiences of Violence

#### Overview

A total of 10 registered nurses working in hospitals in the United States participated in an individual interview. The ethnic and racial breakdown of the participants was: 5 Asian, 2 Latina, 2 African American, and 1 Middle Eastern nurse. All participants identified as female. During their career, these nurses experienced violence from patients and their family members. The violence was categorized into 2 main types: macroaggressions and microaggressions.

#### Macroaggressions

Macroaggressions are more “overt” forms of violence. Macroaggressions are more blatant, often intentional acts of aggression. This form of violence is more deliberate with a clear intent to cause harm against an individual or group [13]. The types of macroaggressions that were experienced by racial and ethnic minority nurses of this study included physical violence, verbal abuse, and sexual violence.

At least once, 70% (7/10) of the nurses experienced physical violence from patients or patients’ family members, such as being hit or swung at. In addition, 90% (9/10) of the nurses experienced verbal abuse. This included yelling, cursing, threats of violence, negative criticism, bullying, name-calling, and derogatory language. Finally, 40% (4/10) of the nurses experienced sexual violence. They experienced being called inappropriate terms of endearment and receiving sexual comments about their body.

#### Microaggressions

Microaggressions—particularly racial microaggressions—are “subtle insults” or “brief, everyday exchanges that send denigrating messages” to people of color because they belong to a racial minority group [14]. Unlike macroaggressions, microaggressions are more subtle and often unconscious and unintentional comments, interactions, or behaviors that communicate bias to an individual. Within this category, 100% (10/10) of racial and ethnic minority nurses experienced racial microaggressions, which were the most common forms of violence reported. Thus, findings related to microaggressions were highlighted in this study.

### Types of Racial Microaggressions Experienced by Racial and Ethnic Minority Nurses

Participants reported experiencing racial microaggressions from patients such as: questioning or inquiries of ethnic and racial origin, generalizations of identity, demeaning comments, questioning of competence, rejection of care, and sexual harassment (see Table 1).

**Table 1.** Examples of microaggressions experienced by racial and ethnic minority nurses in the hospital setting.

Type of racial microaggressions	Examples and quotes
Questioning of ethnic or racial origin	A patient asking a racial and ethnic minority nurse “Where are you from?” or “No, where are you *really* from?” with an assumption that the nurse was not born in America. [5 Asian, 1 Latina nurse]
Questioning of ethnic or racial origin	Openly guessing what their nurse’s race or ethnicity is shortly after they walk into the room. (ie, “Are you Chinese? Japanese? Korean?”) [2 Asian nurses]
Questioning of ethnic or racial origin	A patient relating their racial and ethnic minority nurse to another person they are reminded of due to similar skin color or uniqueness of name. (ie, “Oh, I know another Filipino person!”) [1 African American, 1 Asian nurse]
Generalizations and assumptions of identity	Angry black woman stereotype – Pediatric patients referring to African American nurses as “mean” and White nurses as “nice.” [2 African American nurses]
Generalizations and assumptions of identity	A patient stating, “I want a nurse who was educated in the United States” when they see a minority nurse, or when the nurse has an accent. [2 Asian nurses]
Generalizations and assumptions of identity	“Oh my gosh, you must be so lucky you’re here. You’re very blessed to be in America.” [1 Middle Eastern nurse]
Demeaning or invalidating comments	A patient telling a nurse of Iraqi background about their experiences in the military and associating the country with war and violence. (ie, “Things are so bad [in Iraq] ... It’s very uncivilized.”) [1 Middle Eastern nurse]
Demeaning or invalidating comments	A patient mixing up two nurses of the same race/ethnicity and saying, “You two look alike.” [1 Asian, 1 African American nurse]
Questioning of competence	A patient questioning a racial and ethnic minority nurse more than their White counterparts due to perceived incompetence. (ie, Directing simple questions such as “Do you know what heparin does?” to a minority nurse, but not to their White counterparts) [1 African American, 2 Asian, 2 Latina, 1 Middle Eastern nurse]
Rejection of care	A patient stating, “You’re not going to be my nurse” and requesting a White nurse instead. [2 Asian, 2 Latina nurses]
Sexual harassment	Being called “exotic.” [1 Latina nurse]

### Feelings Behind Microaggressions Among Racial and Ethnic Minority Nurses

All participants (10/10) expressed that they consider microaggressions very harmful. In fact, they considered it to be a form of direct violence. Participants expressed feelings of annoyance, frustration, sadness, discomfort, and anger when experiencing microaggressions.

Racial and ethnic minority nurses disapproved of microaggressions involving questioning or inquiries of ethnic and racial origin as they felt that they were “weird and uncomfortable conversations.” They believed that their identity was not relevant to patient care:

I come in, and I’m like, “Hi, I’m your nurse tonight. Let’s talk about the plan for tonight,” right? And they [the patient] ask me where I’m from… I’m like, “…That’s not the topic at hand.” I’m always like, “Why are we talking about this?”

[Participant A, Asian]

It makes me a little uncomfortable when people are like, “Where are you from?” It annoys me ‘cause… that’s not what you should be thinking about. I’m your nurse. We should be thinking about medical care… not my ethnic background. I know most people are just trying to make small talk and be friendly… It’s just not how I like the small talk to go.

[Participant B, Asian]

Racial and ethnic minority nurses also expressed that when experiencing microaggressions, they often felt that they were only seen “at face value” because of the color of their skin, rather than being seen and accepted as a nurse or as a person:

It makes me view it as like, “Oh, they don’t see me as, like, a true person. They just see me as, like… an identity,” you know? Like, just… the ethnicity. They don’t really take into account, like… I’m my own individual person. They just see color. Black. And they roll with it.

[Participant I, African American]

### Impacts of Microaggressions

Microaggressions were found to have an impact on both a personal and interpersonal level.

#### Impact on Self

On a personal level, participants expressed that microaggressions negatively impact their confidence and self-esteem. The assumptions that patients had made about racial and ethnic minority nurses (ie, being perceived as “mean” or “incompetent”) caused them to “feel inferior from the get-go.” A participant stated,

Why does the way I look have to… attract all of these negative interactions with people? It makes me feel not as worthy, and… not as competent. It just feels like I’m always on edge about my comfort and safety.

[Participant J, Middle Eastern]

As a result of these feelings, nurses of underrepresented minority groups frequently mentioned feeling pressure to “overcompensate.” A participant explained that “as a person of color... you have to work… ten times as hard just to be perceived as more competent in the workplace. You have to make your stance known.” Another participant shared about the challenges she had experienced when trying to build positive relationships with patients:

I had to convince her that I was a nice person instead of that already being assumed. I had to convince her that I was knowledgeable. It’s like you always have to convince people that you are these qualities that they assume of others – or at least, that’s how it feels. I don’t know if that’s just… an insecurity that some people of color have… but I definitely feel like I’ve overcompensated a lot by trying to… prove myself when I shouldn’t have to.

[Participant J, Middle Eastern]

Participants also reported feeling vulnerable as racial and ethnic minority nurses. Although WPV is an issue that affects nearly all health care professionals, nurses of underrepresented backgrounds felt especially prone to violence as they believed that their racial and ethnic background added an “extra layer of vulnerability” that gave patients “something else to target.”

#### Impact on Nurse-Patient Relationship

On an interpersonal level, participants felt that microaggressions impacted their job performance and ruined the nurse-patient relationship. They felt more detached, less willing to engage in conversation, and less willing to go above and beyond when providing care. As a result, they would “minimize staying in the room” by doing the “bare minimum” and “just go in and out.” Participants believed that minimizing their interactions with patients would provide patients with fewer opportunities for microaggressions. A participant explained:

I feel like it [microaggression] is so demeaning. It… ruins the patient-nurse relationships. I don’t do anything bad – It’s just the way I approach them is just different… More detached. I do what I need to do, and I get out. It’s unfortunate because they don’t get the best care that I can give them because they’re being inappropriate.

[Participant G, Latina]

In addition, the nurses often experienced a sense of “vigilance” or anxiety when approaching conversations due to fear of what the patient would say in regard to their racial or ethnic identity. Some explained it as being in a constant state of “fight or flight”:

I think that it [microaggression] has affected the way that I… relate to my patients… There’s that… sort of a barrier inside of me that’s worried that they're gonna be… racist towards me or say something rude and… strike up a conversation that I’m not gonna want to navigate… I think that that affects how I talk to them, which sucks because I don’t want it to… affect the bond that I have with my patients. But sometimes those… anxious feelings kind of do.

[Participant J, Middle Eastern]

### Speaking Up About Microaggressions

Only one (n=1) racial and ethnic minority nurse expressed speaking up about microaggressions to the charge nurse when they occurred, while others remained silent. There were two main reasons behind not speaking up: (1) minimization and (2) normalization.

#### Minimization

Many participants did not speak up about microaggressions to either the perpetrator or management because they felt that their experiences with microaggressions would be undermined or dismissed. Participants felt that they would be seen as “dramatic” if they were to bring these issues to light:

I think because it’s not, like, a “direct” attack, then it’s harder for other people to understand because they’d be like, “Oh, what’s the problem? They just asked you this” or “They were just commenting on their experience with this.”

[Participant H, African American]

#### Normalization

Majority of participants (n=8) of participants expressed that they experienced racial microaggressions not only in the hospital but also in their personal lives outside of work and while growing up. They had become “used to it,” and, as a result, learned to “just deal with it” over time. Thus, they internalized their experiences:

We’re just so used to getting asked that question… ‘cause we don’t wanna focus on it, we just brush it aside… We just… shove it under the rug, ignore it, and that’s what we’re used to doing. And that’s probably why it’s been normalized because people are like, “Oh, they don’t mind being asked that question.” … But the reason that we do it is because we want to move past that… Other people don't see it the same way.

[Participant A, Asian]

### Perceived Causes of Microaggressions Among Racial and Ethnic Minority Nurses

Participants believed that racial microaggressions stemmed from the media, news, and politics, a lack of diversity in the health care team, patients’ lack of exposure to diverse cultural groups, patients’ upbringing, and patients’ inability or unwillingness to unlearn certain stereotypes and assumptions.

### Lack of Support for Racial and Ethnic Minority Nurses

In total, 9 out of the 10 interviewed participants (n=9) expressed that they do not feel supported in the hospital as nurses of underrepresented minority groups due to three main reasons: (1) lack of conversations and acknowledgment surrounding the unique struggles that racial and ethnic minority nurses face, (2) lack of resources on diversity, equity, and inclusion, and (3) lack of diversity within the health care team:

I feel like it hasn’t even been brought up… not really any topics regarding… diversity, or the fact that we’re [racial and ethnic minority nurses] kind of more vulnerable to WPV… I think that they don't acknowledge that it’s there, which is the bigger issue.

[Participant H, African American]

I… don’t have anybody on the unit that is… Latina as well to… talk about these things… So, I do feel alone sometimes in that sense.

[Participant F, Latina]

## Discussion

### Principal Findings

Microaggressions were highlighted in our study. All participants in our sample (10/10) experienced racial microaggressions by patients in the hospital, and they considered microaggressions as a form of WPV. Many racial and ethnic minority nurses referred to their accounts of microaggressions as “little comments” or “small comments” that may not be seen as a form of violence in others’ eyes, especially when comparing the magnitude of this violence to more blatant forms of violence such as physical attacks or sexual assault. Microaggressions are falsely believed to have minimal negative impact, and people of color are often told not to “overreact” and simply “let it go.” [14] This was a common sentiment among nurses from racial and ethnic minority groups in this study.

However, even though the prefix “micro” means “small,” all interviewed participants expressed that they consider microaggressions very harmful. Microaggressions had a negative impact on participants’ confidence and self-esteem, causing feelings of inadequacy and inferiority. Participants felt that they had to work harder to prove their competence in the workplace. Microaggressions unfortunately impacted the nurse-patient relationship as well. Participants experienced decreased job performance and felt more detached from their work after experiencing microaggressions. They limited interactions with patients and spent less time in patients’ rooms due to anxiety, ultimately compromising the quality of care that was delivered. Previous studies have shown that microaggressions can be “detrimental to persons of color because they impair performance in a multitude of settings by sapping the psychic and spiritual energy of recipients and by creating inequities.” [14] This shows that microaggressions are subtle, but the consequences are additive and prevent the delivery of adequate, patient-centered care [15].

Most nurses (n=9) did not feel supported in the hospital. They expressed that there was a lack of conversations and acknowledgment regarding the fact that racial and ethnic minority nurses can be more vulnerable to WPV. They wished for more resources and topics that teach about diversity, equity, and inclusion, as well as more diverse health care teams.

### Comparison With Previous Work

The findings of this study were consistent with those of another study that explored racial microaggressions experienced by minority registered nurses working in a hospital in the mid-Atlantic region. Microaggressions from patients and their families were commonly experienced by racial and ethnic racial and ethnic minority nurses, and examples included biased first impressions (ie, making comments or assumptions about race), “firing” the nurse (ie, refusal of care due to race), and questioning experience or credentials [16]. Nurses of this study also “felt that they had to prove themselves to be perceived as equal to their White coworkers,” [16] which was a similar finding among nurses of our study.

Racial microaggressions are a common experience among individuals from racial and ethnic minority groups, and they are so “pervasive and autonoetic in daily conversations and interaction that they are often dismissed and glossed over as being innocent and innocuous.” [14] However, there are several research studies that have shown the detrimental effects of racial microaggressions in the health care setting [17].

In a cross-sectional, correlational study, racial and ethnic minority nurses reported experiencing racial microaggressions at a rate that was nearly 3 times higher than their White counterparts, and racial microaggressions had a large effect on the likelihood of severe emotional distress [18]. Another study explored the specific impact of racial microaggressions among underrepresented medical and nursing students at various universities in the United States. Overall, students felt devalued by racial microaggressions and experienced stress, frustration, and anger; they also believed that microaggressions negatively impacted their academic performance and personal well-being [19]. In a study that examined medical students’ experiences with microaggressions, the cumulative effects were devastating—the results were feelings of self-doubt, isolation, poor academic and learning performance, impaired productivity, well-being, and mental function, and weakened relationships [20]. These findings are congruent with those of our study, further supporting the idea that microaggressions are both harmful and cumulative.

In our study, racial microaggressions and WPV were shown to impact not only the nurses but also the delivery of patient care. In one study, health care providers who experienced WPV in the emergency department were interviewed and asked to identify the consequences of type II WPV [21]. Participants believed that while health care providers experienced the most consequences, patients and patient care suffered greatly as well. In fact, participants reported that fear of violence often affected their medical decision-making, causing them to treat patients in the manner least likely to result in a violent outcome, rather than doing what is medically indicated. These findings align with those of our study, where participants felt that limiting their interactions with patients or spending less time in patients’ rooms would reduce their exposure to microaggressions. Other studies have reported that type II WPV and discrimination are correlated with burnout among health care professionals, and burnout results in higher odds of major medical errors [22].

Racial and ethnic minority nurses in this study expressed that they do not feel supported in the hospital. More specifically, they reported inadequate support from management in terms of navigating microaggressions, which fuels the normalization of microaggressions in the hospital setting. Other studies have shown that many faculty educators lack formal training in supporting students after incidents of microaggressions in the clinical environment, especially since microaggressions are more difficult to recognize or easily dismissed compared to more overt forms of WPV and macroaggressions [23]. This highlights the need for strategies, resources, and structural accountability to address and mitigate microaggressions in the clinical setting. Examples of structural accountability include implicit bias and antiracism training (ie, set policies, training tools when microaggressions are witness or experienced) and identification of mentors to provide a support system where recipients of microaggressions feel protected and valued [24].

Most of the nurses in our study did not speak up about microaggressions after they occurred to either the perpetrator or to management. Sue et al [25] outlines four key strategies for victims, allies, and bystanders when addressing microaggressions: (1) make the “invisible” visible by raising awareness to the fact that microaggressions do exist, (2) disarm the microaggression by immediately stopping it, interrupting or redirecting, communicating disagreement, stating values, and setting limits, (3) educate the offender on how or why their microaggression is harmful, and (4) seek external support by alerting leadership, reporting the act, and creating community with friends, allies, and support groups. At the systemic level, hospitals can offer workshops centered around educating employees on microaggressions. There is one study that implemented an active bystander training workshop with physicians; the workshop used cased-based simulations with standardized patients to teach physicians how to respond to microaggressions. Participants of this workshop reported statistically significant improvement in recognizing microaggressions, responding to patient’s microaggressions, and debriefing with team members [26]. Other workshops incorporating educational PowerPoints and small-group discussions have been reported to have high satisfaction from participants as well [27,28].

Many of our participants expressed the importance of diversifying the health care team. Studies have shown that there is a lack of underrepresented minorities in health care leadership positions. In nursing specifically, only 20% of nurses are of racial and ethnic groups [29]. Since it can be easier for marginalized groups to recognize microaggressions than nonmarginalized groups, promoting diversity in the health care setting would improve the recognition of microaggressions, eventually leading to the implementation of institutional policies and culture change [23]. In this study, racial and ethnic minority nurses reported feeling supported and greater job satisfaction when working with other minorities. In addition, they expressed a sense of hesitancy in speaking up about their experiences to their White coworkers due to fear that their concerns would be undermined. A participant expressed that they felt alone and isolated in their experiences as they lacked other racial and ethnic minority nurses to turn to. Similarly, one study found that when encountering WPV, racial and ethnic minority nurses often sought support from the larger network of nurses from racial and ethnic minority groups at the hospital as a way to cope with conflicts [10].

Hospitals can diversify health care teams through various strategies. First and foremost, diversity and inclusion should be ingrained within the culture of the organization as a whole by making these values an integral part of its mission [29]. In addition, stakeholders from all levels of the organization should be included in discussions regarding diversity, equity, and inclusion so that these values are upheld at every structural level [29]. Given this, increased racial and ethnic minority nurse representation in leadership and decision-making roles is critical. A key method in achieving this would be to provide mentorship programs that support racial and ethnic minority nurses in obtaining leadership positions [30]. Finally, it is important for recruiters to recognize and eliminate their own biases during the recruitment process through implicit bias training [31].

### Strengths and Limitations

There are several strengths to this study. For one, this study contributes to the gap in the literature that exists regarding the experiences of racial and ethnic minority nurses with WPV, specifically in the context of microaggressions. In addition, the qualitative approach of this study allowed participants to elaborate on their experiences and provide specific details. The sample was also diverse in their racial and ethnic identities. Finally, the research team was composed of individuals who are well-versed in this topic of study. They are racial and ethnic minorities with health care experience, and the second author’s (HDB) research expertise lies in WPV in health care settings. Thus, their background and knowledge allowed for the study to be strategically designed in a way that is not only culturally sensitive but also meets the research aims.

A limitation of this study is the lack of previous research on this topic which prevented us from replicating procedures or building on significant findings. In addition, because our interviews were not intentionally designed to achieve diversity across other demographics such as gender, our sample consisted of exclusively female participants, which limits the generalizability of findings. The snowball sampling method and small sample size make it challenging to make accurate inferences about the larger population as well. It is also important to acknowledge that while the semistructured interviews allowed for more fluidity in conversation, they may have resulted in inconsistencies in data collection. For example, valuable questions that were posed to some participants were not posed to other participants at times.

### Future Research

More research can be done to explore variability in WPV experiences across different racial and ethnic groups. For example, a study found that Asian American nurses experienced an increase in discrimination after COVID-19, and 75% of nurses experienced job harassment, unfair treatment, and feeling invisible at work [32]. Only 1 study exploring WPV among Hispanic nurses was found. The study found that some of the top reported WPV events were emotional-verbal types of violence such as “criticized,” “made to feel bad,” “shouted or yelled at,” and “insulted or sweared at.” Sexual types of WPV were also some of the most common among Hispanic nurses, including “suggestive looks” and “sexist remarks” [33]. Another study that explored mistreatment in the workplace among physicians of various racial and ethnic groups found that verbal mistreatment was the highest among physicians from Black communities and lowest among physicians from White communities [34]. Physicians from multiracial and Black communities were more likely than physicians from White and Asian communities to report experiencing at least one form of mistreatment [34]. These findings highlight the need for further research to better understand if and how WPV manifests differently across various minority groups.

In addition, studies are needed to explore the role that intersectionality plays in WPV. Although this study highlights how race and ethnicity impacts WPV, it is important to acknowledge that other aspects of an individual’s identity—gender, age, sexuality, disability, class, immigration status, religions, cultural background, and other social and demographic identities—may shape a unique experience. For example, while sexual harassment impacts all individuals, research has shown that lesbian women and women who do not conform to traditional feminine expectations are more often targets of sexual harassment, and older women have been found to face more discrimination than older men based on physical appearance [35]. The study also found that 69% of women wearing a hijab have experienced at least one incident of discrimination [35]. In our study, all participants identified as female, which may have impacted our findings in some way. Taking an intersectional approach to future WPV studies is a complex yet necessary step to better understand individuals’ lived experiences.

There is a lack of literature on effective interventions for confronting and preventing microaggressions in health care settings. More research is needed to examine successful models that tackle microaggressions, as well as institutional efforts aimed at supporting racial and ethnic minority nurses. This would allow hospitals to implement similar interventions at their own facilities.

Quantitative studies examining the long-term effects of microaggressions on racial and ethnic minority nurses’ well-being would provide valuable data as well. Racism-related stress theory [36] suggests that individuals who encounter racism, prejudice, and discrimination also experience high levels of stress, leading to long-term outcomes in 5 domains: physical (ie, hypertension and cardiovascular reactivity), psychological, (ie, general psychological distress and trauma-related symptoms), social (ie, decreased ability to trust or have close relationships), functional (ie, decreased job performance), and spiritual (ie, threatened vitality of spirit and faith). More research is needed to study how racial microaggressions may affect racial and ethnic minority nurses in each of these domains.

At the same time, studies can be conducted to explore how racial microaggressions may empower racial and ethnic minority nurses. One scoping review found several studies reporting that “the experience of racism, though challenging, also motivated participants to succeed or overcome obstacles, suggesting a complex interplay between adversity and resilience among minority nurses” [37]. Racial and ethnic minority nurses in this study expressed experiencing microaggressions not only in the hospital but also in their day-to-day personal lives and while growing up. To have continued to maintain their dignity in the face of hostility is a testament to their resiliency [14]. Exploring their coping mechanisms and strategies may empower those who have faced similar experiences.

Finally, studies are needed to explore type III (worker-on-worker) WPV and racial microaggressions. Although not included in the results of this study, participants did mention experiencing racial microaggressions from coworkers. Studying this may highlight the need for increased cultural awareness and education among health care professionals.

### Conclusions

The findings of this study offer insight into the genuine thoughts, feelings, and experiences of nurses from racial and ethnic minority groups who have experienced WPV in the hospital setting. This study shows that microaggressions are a form of WPV. By recognizing microaggressions and understanding their harmful effects, hospitals and management can provide resources that aid racial and ethnic minority nurses in navigating such incidents. In addition, the findings raise awareness of what may constitute microaggressions, which sheds light on the need for patients, family members, and health care professionals to be more educated and culturally aware on issues that impact marginalized communities. Supportive organizational infrastructures need to be in place to enhance diversity awareness and encourage better multicultural interactions in the workplace [38]. Finally, racial and ethnic minority nurses thrive in diverse health care teams—prioritizing diversity and inclusion in the workplace can improve feelings of support, safety, and self-esteem among the nurses as well as cultivate a culturally sensitive environment. Addressing these issues will improve the nurse-patient relationship and the delivery of care.

## Supplementary material

10.2196/67029 Multimedia Appendix 1 Semistructured interview guide used during the data collection process.

## Abbreviations

WPV: workplace violence
