# Supplementary material for: The Lived Experiences of Racial and Ethnic Minority Nurses Exposed to Racial Microaggressions in the Hospital Setting: Qualitative Study
Source: Asian Pac Isl Nurs J. 2025 Jun 18;9:e67029. doi: 10.2196/67029 (PMC12192909; doi:10.2196/67029)
Supplement: Multimedia Appendix 1 [file apinj-v9-e67029-s001.docx]

**Semi-Structured Individual Interview Guide**

**PURPOSE:**

The purpose of this study is to (a) explore the lived experiences of racial and ethnic minority nurses who have faced type II WPV from patients in the hospital setting, and (b) assess the emotional and physical effects of type II violence among minority nurses.

**INTERVIEW QUESTIONS/PROMPTS:**

- **Introduction**
  - Describe the study, PI’s role, risks and benefits, and contents to participants
- **Opener questions**
  - What race/ethnicity best describes you?
    - African American, Latino, Asian/Native Hawaiian, American Indian/Alaska Native, Middle Eastern, multiracial, prefer not to say
  - Tell me about your job.
  - Tell me what your typical work day looks like.
  - Tell me about your interactions with your patients, their family members, or visitors.
- **Experiences with workplace violence**
  - Tell me about any situation where you experienced workplace violence from patients, their family members, or visitors during your time as a nurse. Violence is defined broadly including, but not limited to: verbal abuse (demeaning comments, racist remarks, yelling, cursing), physical assaults/attacks, sexual assaults/harassment, bullying, etc.
- **Thoughts, feelings, perceptions**
  - How did you feel when you experienced this workplace violence?
  - What thoughts occur to you when you experience workplace violence?
  - Did you speak up to anyone about your experiences with workplace violence? Why or why not?
  - In what ways, if any, does workplace violence affect your job performance?
  - Do you ever feel that your experiences in the workplace are correlated to your racial/ethnic identity?
  - As a racial/ethnic minority nurse, in what ways do you feel supported or not supported in your work environment?

**Questions to potentially ask to follow up on questions above:**

- Can you tell me more about that?
- Can you elaborate on that?
- How did you feel about that?
